# The predominant lactic acid bacteria and yeasts involved in the spontaneous fermentation of millet during the production of the traditional porridge *Hausa koko* in Ghana

**DOI:** 10.1186/s12866-024-03317-1

**Published:** 2024-05-14

**Authors:** Amy Atter, Maria Diaz, Kwaku Tano-Debrah, Angela Parry-Hanson Kunadu, Melinda J. Mayer, Lizbeth Sayavedra, Collins Misita, Wisdom Amoa-Awua, Arjan Narbad

**Affiliations:** 1 Food Microbiology and Mushroom Research Division, CSIR-Food Research Institute, Accra, Ghana; 2 Department of Nutrition and Food Science, University of Ghana, Accra, Ghana; 3 Food and Health Institute Strategic Programme, Quadram Institute Bioscience, Norwich Research Park, Norwich, UK; 4 Gut Microbes and Health Institute Strategic Programme, Quadram Institute Bioscience, Norwich Research Park, Norwich, UK; 5 Department of Biochemistry, Cell and Molecular Biology, University of Ghana, Accra, Ghana; 6 Department of Agro-Processing Technology and Food Bio-Sciences, CSIR College of Science and Technology, Accra, Ghana

**Keywords:** Lactic acid bacteria, Yeast, Fermented millet, Whole genome sequencing (WGS), *Hausa koko*

## Abstract

Spontaneous fermentation of cereals like millet involves a diverse population of microbes from various sources, including raw materials, processing equipment, fermenting receptacles, and the environment. Here, we present data on the predominant microbial species and their succession at each stage of the *Hausa koko* production process from five regions of Ghana. The isolates were enumerated using selective media, purified, and phenotypically characterised. The LAB isolates were further characterised by 16S rRNA Sanger sequencing, typed using (GTG)_5_ repetitive-PCR, and whole genome sequencing, while 28S rRNA Sanger sequencing was performed for yeast identification. The pH of the millet grains ranged from mean values of 6.02—6.53 to 3.51—3.99 in the final product, depending on the processors. The mean LAB and yeast counts increased during fermentation then fell to final counts of log 2.77–3.95 CFU/g for LAB and log 2.10–2.98 CFU/g for yeast in *Hausa koko* samples. At the various processing stages, the counts of LAB and yeast revealed significant variations (*p* < 0.0001). The species of LAB identified in this study were *Limosilactobacillus pontis*, *Pediococcus acidilactici*, *Limosilactobacillus fermentum*, *Limosilactobacillus reuteri*, *Pediococcus pentosaceus*, *Lacticaseibacillus paracasei*, *Lactiplantibacillus plantarum*, *Schleiferilactobacillus harbinensis*, and *Weissella confusa*. The yeasts were *Saccharomyces cf. cerevisiae/paradoxus*, *Saccharomyces cerevisiae*, *Pichia kudriavzevii, Clavispora lusitaniae* and *Candida tropicalis*. The identification and sequencing of these novel isolates and how they change during the fermentation process will pave the way for future controlled fermentation, safer starter cultures, and identifying optimal stages for starter culture addition or nutritional interventions. These LAB and yeast species are linked to many indigenous African fermented foods, potentially acting as probiotics in some cases. This result serves as the basis for further studies into the technological and probiotic potential of these *Hausa koko* microorganisms.

## Introduction

Cereal crops including rice, maize, wheat, rye, barley, millet, and sorghum are produced globally and considered an important and good source of carbohydrates, dietary proteins, irons, trace minerals, fibre, and vitamins [1]. These cereals are a good substrate for the growth of both beneficial and detrimental microorganisms. They have been described as functional foods because they contain sufficient quantities of biologically active components that are capable of imparting health benefits to the consumer in addition to the nutrients they provide [2–6]. Contrary views have also been expressed about them, as they are sometimes considered inferior due to their deficiency in some essential amino acids, resulting in lower protein quality compared to some other crops, and the presence of anti-nutritive compounds including tannins, phytic acid, and phenols [7, 8]. Foods prepared from unfermented cereals have also been described as lacking flavour and aroma [4].

Cereals in their dried states are metabolically inactive, including their enzymes. However, when they absorb water, for example during steeping, their enzymes are activated, leading to the hydrolyzation of macromolecules and initiation of spontaneous fermentation through the growth and proliferation of contaminating microorganisms [4]. Such fermentation processes have been used to overcome their initial nutritional limitations and fermented cereals are considered superior due to the functional properties of the key fermenting microorganisms involved [1, 3, 4, 9].

The microbial ecology of fermented cereals such as millet, used in the production of many indigenous foods in Africa, mostly involves a mixed population of microbes. One such indigenous food is *Hausa koko,* a spicy, smooth, and free-flowing fermented pearl millet porridge produced in Ghana. *Hausa koko* is commonly sold as a hot street food in Ghana and it plays a significant role in contributing to food security because it is available, accessible and affordable. *Hausa koko* production involves steeping of millet grains for 12–24 h after which it is washed, milled with spices, and the resulting flour is mixed with water to form a slurry. The slurry is sieved and allowed to ferment for 8—12 h during which it separates into supernatant and sediment. Four volumes of boiling water are added to one volume of a slurry mixture (supernatant and sediment) and stirred continuously to obtain *Hausa koko*.

We have previously described the microbial ecology of *Hausa koko* using amplicon sequencing [2]. The analysis revealed a diverse range of Gram-positive and Gram-negative microorganisms and yeasts including *Staphylococcus, Enterobacteriaceae, Pseudomonas, Sphingomonas, Clostridium, Leuconostoc, Gluconobacter, Streptococcus, Escherichia-Shigella, Kluyveromyces, Nakaseomyces, Torulaspora,* and *Cyberlindnera*. These microorganisms are associated with the soil, raw material, environment, and production process [2]. The mixed population, however, reduces during the spontaneous fermentation with an increase and predominance of lactic acid bacteria (LAB) and yeasts [2, 4, 5, 10]. In studies of spontaneously fermented sour products in Africa, yeasts have mostly been reported to play a key role in the fermentation alongside the LAB, which are responsible for the souring of the product. The yeasts are reported to facilitate the growth of the LAB and also contribute to the flavour of the product [11, 12]. A combination of phenotypic and high throughput Next Generation Sequencing methods that have high discriminatory power, accuracy, and sensitivity can be used to provide comprehensive information about these key fermenting microorganisms. The presence of various LAB and yeast species has been reported in other African fermented foods [13–26].

In the present study, whole genome and Sanger sequencing were employed respectively for an in-depth description of the lactic acid bacteria and yeasts involved in the spontaneous fermentation of millet into the traditional millet porridge, *Hausa koko,* in Ghana*.* Such an approach to the study of microbial isolates from traditional fermentation processes not only identifies the fermenting microorganisms but also indicates their functionality, facilitating the selection of beneficial specific traits for commercial exploitation including the development of starter cultures to upgrade the traditional processes for adoption by Small and Medium Scale Enterprises. It also allows undesirable traits such as virulence factors or antimicrobial resistance genes to be avoided in developing the starter culture.

## Materials and methods

### Sampling

Samples were collected from various stages of *Hausa koko* fermentation by traditional food processors from a total of five (5) production sites from 5 out of the sixteen (16) political regions of Ghana. These were Tamale Dabokpa (TAD) in the Northern Region, Sunyani (SUN) in the Bono Region, Mankessim (MAN) in the Central Region, Dodowa (DOD) in the Eastern Region, and Accra Madina Zongo (AMZ) in the Greater Accra Region. The samples collected at each production site were millet grains (D), steeped millet grains (at the end of the steeping process, either 12 or 24 h depending on the processor), milled steeped millet with spices (M), fermented slurry—supernatant (Su), fermented slurry- sediment (Sd), and *Hausa koko* (K). They were collected aseptically into sterile sampling containers and transported to the CSIR-Food Research Institute in Accra under cold storage where they were preserved at -20 °C. Samples were then transported under cold storage to the Quadram Institute Bioscience (QIB), Norwich, UK for analysis.

### Microbiological analysis

One gram (1 g) of the sample was added to 9 ml of sterile phosphate-buffered saline (PBS) solution with pH adjusted to 7.2 and vortexed for 30 s at normal speed. Ten-fold dilutions were prepared and 100 µl each dilution were inoculated into the appropriate selective media for enumeration and isolation of lactic acid bacteria and yeasts. The spread plate method was used in the enumeration of Lactobacilli using deMan, Rogosa, and Sharpe (MRS, Oxoid CM359, Oxoid Ltd., Basingstoke, Hampshire, UK.) with 1.5% agar (AGA03, Formedium Ltd, UK) adjusted to pH 6.2. The media was supplemented with 0.1% cycloheximide (A0406195, Acros Organics, China) to inhibit the growth of yeast and incubated aerobically at 37 °C for 2–3 days. For the enumeration of *Lactococcus* species, M17 (Oxoid CM 0817, Oxoid Ltd., Basingstoke, Hampshire, UK.) supplemented with 0.5% lactose and 1.5% agar was used. Enumeration of yeast was performed by the spread plate method using Rose Bengal Chloramphenicol Agar (Oxoid CM 0549 Oxoid Ltd., Basingstoke, Hampshire, UK) pH 5.5. The plates were incubated at 25 °C for 3–5 days. Ten colonies of LAB and yeast were selected from each segment of the highest dilution or appropriate MRS, LM17 (for lactic acid bacteria), or Rose Bengal (for yeast) plate and streaked repeatedly on the appropriate agar plate until pure colonies were obtained.

We characterised phenotypically the LAB pure cultures on MRS plates based on their colony morphology. Using a validated in-house method by CSIR-FRI, catalase activity was determined by emulsifying a pure single bacterial colony on a slide containing 3% hydrogen peroxide for the liberation of bubbles or free oxygen, while oxidase activity was determined using oxidase test strips (Oxoid Limited, Basingstoke, Hampshire, UK) [27]. Gram staining was performed using a Gram staining kit (Remel, Thermo Fisher Scientific, USA). The cell morphology of the Gram-stained slides was examined under a phase contrast microscope (Olympus BX60F5, Japan).

For the characterisation of yeast isolates, the colony morphology of the isolates was determined on Rose Bengal Chloramphenicol Agar using size, colour (pink, cream, white, off-white), surface (smooth, smooth and shiny, hirsute), appearance (elongated, ovoid, globose), elevation (raised, umbonate, concave), and margin (entire, filiform or wavy) as parameters. Growth patterns of yeast in liquid medium including sedimentation, gas production, pellicle formation between glass and liquid interphase, and turbidity were examined in 20 mL Yeast Mold broth, YM (BD 271120, Becton, Dickinson, USA) in bijou bottles as described by [28].

### Molecular identification and typing of LAB and yeast isolates

LAB isolates were identified using the 16S rRNA while yeasts were identified using the D1/D2 region of the 28S rRNA. To amplify the respective fragments, PCR reactions were set up from 150 μL overnight cultures grown in broth medium; cultures were centrifuged for 1 min at 13,000 × g, washed with 150 μL colony wash buffer (100 mM NaCl,10 mM Tris–HCl pH 7, 1 mM EDTA), re-suspended in 15 μL ultra-pure H_2_O and heated at 95 °C for 5 min. The PCR reactions were performed in a thermal cycler (Biometra GmbH, Germany). For bacterial identification, the universal primers AMP_F (5’ GAGAGTTTGATYCTGCGCTCAG 3’) and AMP_R (5’ AAGGAGGTG ATCCARCCGCA 3’) were used for the amplification of the 16S rRNA genes according to Baker et al*.,* (2003), [29] while primers NL1 (5’ GCATATCAATAAGCGGAGGAAAAG 3’) and NL4 (5’ GGTCCGTGTTTCAAGACGG 3’) [30] were used for yeast identification. The amplification for primers AMP_F/AMP_R was conducted at 95 °C initial denaturation for 2 min, followed by 25 cycles of 95 °C denaturation for 30 s, 55 °C annealing for 30 s, 72 °C extension for 1 min, a final extension at 72 °C for 5 min, giving a c. 1.5 Kb product. Amplification for primers NL1/NL4 was performed with an initial denaturation at 94 °C for 5 min, followed by 25 cycles of 92 °C denaturation for 30 s, 54 °C annealing for 30 s, 72 °C extension for 1 min/kb, and final extension at 72 °C for 5 min. The resulting amplicons were visualized in 1% agarose gels.

28S rRNA gene sequencing was performed using purified yeast PCR products by Eurofins, UK. Sequenced read sets from the yeast isolates were assembled and manually revised using EditSeq v 5.06 and SeqMan II v 5.06 software packages (DNASTAR. Inc). The assembled sequences were identified using the Ribosomal Database Project (RDP) using typed strains only to identify isolates to the species level. GenBank accession numbers are from OR186448—OR186505.

The LAB isolates were typed using Rep-PCR with the primer GTG5 (5’ GTGGTGGTGGTGGTG 3’) [31] with the purpose of selecting isolates for whole genome sequencing. The amplification was programmed at 94 °C initial denaturation for 4 min, followed by 30 cycles of 94 °C denaturation for 30 s, 45 °C annealing for 1 min, 72 °C extension for 8 min and final extension at 72 °C for 16 min. Amplicons were separated by electrophoresis run at 115 V for 5 h 30 min in a 1% agarose gel.

### Whole genome sequencing

Genomic DNA extraction was performed using a 96 well plate DNA extraction method for LAB according to the method described by [32] with the following modifications. Each plate well contained 50 µL of the cell suspension and 100 µL of lysing buffer (0.02 g lysozyme, 10 mL TE buffer, 100 µL RNAse A (10 mg/mL) and 100 µL Mutanolysin (10 KU/mL). The wells were placed on a thermomixer set to 37 °C and shaken at 1600 rpm for 30 min. 10 µL of lysing additive (528 µL TE buffer, 600 µL 10% SDS buffer, 60 µL of 20 mg/mL Proteinase K and 12 µL RNAseA) were added to each well, re-suspended and placed on a thermomixer set to 65 °C 1600 rpm for 15 min. About 100 µL of the suspension was pipetted from the wells to a new lo-bind PCR 96 well plate for DNA purification using solid-phase reversible immobilisation magnetic beads (AMPure XP, Beckman Coulter Inc, USA). The magnetic beads (50 µL) were added to each well, mixed and incubated at room temperature for 5 min. The plate was placed on a magnetic instrument and left for 5 min to settle. The supernatant was removed and the beads were washed three times with 100 µL of freshly prepared 80% ethanol which was subsequently removed. The plate was allowed to dry off for 2 min, taken off the magnetic apparatus and DNA eluted from the beads using 50 µL 10 mM Tris–HCl (pH 8).

Following manufacturer instructions, the Qubit 3.0 fluorometer (Invitrogen, Malaysia) was used to measure DNA concentrations using dsDNA Broad Range (BR) and dsDNA High Sensitivity (HS) assay kits and gDNA was stored at -20 °C until ready for sequencing. Whole genome sequencing of the LAB isolates was conducted at the Earlham Institute (Norwich, UK). The gDNA extracted from pure cultures was used to construct low-input transposase enabled (LITE) libraries. Libraries were sequenced using the Illumina HiSeq4000 platform with 150 bp paired-end reads.

### Genome assembly and phylogenetic analyses

To assemble the genomes of the bacterial isolates, the short reads were first taxonomically classified with centrifuge v. 1.0.3 (https://ccb.jhu.edu/software/centrifuge) using as reference the NCBI database [33]. Classified reads were then filtered with kt extract, contained in the ktoolu software package (https://github.com/cschu/ktoolu) as follows: reads that were classified as fungal were discarded while bacterial and unclassified reads were retained. Adapters were removed, reads were quality trimmed with a minimum quality phred score of 3, and reads with a length below 100 bp or average quality of less than phred 20 were discarded using the bbduk v. 37.24 (https://jgi.doe.gov/data-and-tools/bbtools). Cleaned read sets were normalized to a maximum coverage of 100 with bbnorm v. 37.24. The quality-controlled and normalized reads were assembled with the unicycler-pipeline (unicycler: 0.4.3_cs2, spades: 3.8.1) using the spades-optimizing mode [34]. For the optimization, sample-specific k-mer ranges were determined by unicycler. As part of the pipeline, reads were error-corrected by SPAdes [35] and the resulting contigs polished with pilon v. 1.22 [36]. Assemblies were quality checked with QUAST v. 4.3 [37] and CheckM v.1.2 [38].

Based on the CheckM contamination predictions, 33 isolates were suspected not to be pure. For these samples, we reassembled the metagenomes using Metaspades v.3.11.1 [39]. Metagenome-assembled genomes (MAGs) were obtained using MetaBAT v.2.12.1 [40], using the coverage per scaffold calculated using BBmap v.38.43. The resulting MAGs were quality checked with CheckM and only those with a completeness > 80% and a contamination < 5% were further considered. Genomes were classified taxonomically using GTDB-Tk v.2.1.1 [41]. All genomes were annotated using PATRIC v.3.6.3, which provides subsystem annotation [42]. Genomes and reads have been deposited to NCBI with the accession number PRJNA932444.

For phylogenomic reconstruction, reference genomes were obtained from BV-BRC [43]. For phylogenomic reconstruction, 29 marker genes were extracted with AMPHORA2 [44] and aligned with Muscle v.3.8.31 [45] using the phylogenomic-tools pipeline (https://github.com/kbseah/phylogenomics-tools). The concatenated protein alignment was masked to remove alignment positions with > 75% gaps using Geneious Prime [46] and a tree with 100 rapid bootstrap and subsequent maximum likelihood search was reconstructed with the GAMMA model of rate heterogeneity using RaxML v.8.2.11 [47]. The tree was visualized and edited with iTol [48].

For yeast, the 28S rRNA sequences were aligned with MAFFT v.7.505 (https://mafft.cbrc.jp/alignment/software/) and phylogenetic analysis was performed using RAXML v.8.2.12 (https://academic.oup.com/bioinformatics/article/30/9/1312/238053) [49]. The phylogenetic trees were annotated by the species, the production sites, and the stages using R software v.4.0.2.

### Statistical analysis

Technical replicates of pH measurements and microbial counts were obtained for each sample and mean values calculated. Subsequently, differences in the mean values of pH and microbial counts across various timepoints from all producers were assessed using analysis of variance (ANOVA) with the oneway.test function and the Turkey HSD post-hoc test with the glht function of the multcomp package in R version 4.2.3.

## Results

### Reduction in pH

The pH of the millet grains decreased during the steeping and fermentation/souring of the millet slurry, as observed across all the production sites in the five different regions (*p*-value < 0.0001) (Table 1). The mean pH values of the millet grains ranged from 6.02 to 6.53. During steeping of the millet grains for 12 or 24 h, the mean pH values dropped to 4.35–4.08 (*p*-value < 0.001), after milling of the steeped millet grains together with the spices, the mean pH dropped slightly but no significant differences were found either with the 12 or 24 h steeped samples (*p*-value = 0.1647 and 0.5283 respectively). After fermentation of the millet slurry, the mean pH of the supernatant dropped (*p*-values < 0.001) to 3.27–3.68 and the sediment to 3.23–3.65 to then remain the same in the final product *Hausa koko* (*p*-value = 0.4422 and 0.1378).

**Table 1:** Mean pH and microbial counts (log CFU/g) at various stages of *Hausa koko* production from 5 processors

**Processors**	Stages*	**pH**	**LAB (MRS)**	**LAB (M17)**	**Yeast**
**Tamale**	TAD-D	6.02 ± 0.01^a^	3.18 ± 0.01^c^	1.71 ± 0.01^c^	2.02 ± 0.03^d^
	TAD-12 h	4.08 ± 0.01^b^	7.84 ± 0.02^a^	3.36 ± 0.03^b^	5.76 ± 0.02^a^
	TAD-M	3.91 ± 0.01^c^	-	-	-
	TAD-Su	3.27 ± 0.01^e^	7.76 ± 0.02^a^	3.27 ± 0.02^b^	4.92 ± 0.01^b^
	TAD-Sd	3.28 ± 0.01^e^	7.64 ± 0.02^b^	3.56 ± 0.01^a^	4.97 ± 0.01^b^
	TAD-K	3.65 ± 0.01^d^	2.77 ± 0.02^d^	1.49 ± 0.02^d^	2.27 ± 0.02^c^
**Sunyani**	SUN-D	6.53 ± 0.01^a^	3.45 ± 0.03^d^	1.97 ± 0.02^c^	2.26 ± 0.01^e^
	SUN-12 h	4.33 ± 0.01^b^	-	-	-
	SUN-24 h	4.31 ± 0.01^b^	8.99 ± 0.01^a^	4.50 ± 0.02^a^	5.74 ± 0.01^b^
	SUN-M	4.07 ± 0.01^c^	-	-	-
	SUN-Su	3.43 ± 0.01^e^	7.79 ± 0.01^c^	2.70 ± 0.01^b^	5.51 ± 0.01^c^
	SUN-Sd	3.35 ± 0.01^f^	7.97 ± 0.01^b^	2.73 ± 0.01^b^	5.89 ± 0.01^a^
	SUN-K	3.51 ± 0.01^d^	3.19 ± 0.02^e^	1.70 ± 0.01^d^	2.98 ± 0.02^d^
**Mankessim**	MAN-D	6.14 ± 0.01^a^	4.79 ± 0.01^d^	3.64 ± 0.02^d^	2.81 ± 0.01^d^
	MAN-12 h	4.59 ± 0.01^b^	-	-	-
	MAN-24 h	4.35 ± 0.01^d^	8.86 ± 0.01^b^	5.82 ± 0.01^a^	6.65 ± 0.01^b^
	MAN-M	4.42 ± 0.01^c^	-	-	-
	MAN-Su	3.43 ± 0.01^e^	8.74 ± 0.02^c^	5.68 ± 0.02^b^	6.54 ± 0.02^c^
	MAN-Sd	3.35 ± 0.01^f^	8.94 ± 0.01^a^	5.88 ± 0.01^a^	6.98 ± 0.01^a^
	MAN-K	3.95 ± 0.01^d^	3.95 ± 0.01^e^	3.93 ± 0.01^c^	2.57 ± 0.01^e^
**Dodowa**	DOD-D	6.27 ± 0.01^a^	3.95 ± 0.01^c^	3.59 ± 0.01^b^	3.88 ± 0.01^d^
	DOD-12 h	4.41 ± 0.01^b^	7.72 ± 0.01^b^	4.87 ± 0.01^a^	5.24 ± 0.02^a^
	DOD-M	3.98 ± 0.01^c^	-	-	-
	DOD-Su	3.58 ± 0.01^d^	8.93 ± 0.01^a^	2.76 ± 0.01^d^	4.80 ± 0.01^b^
	DOD-Sd	3.38 ± 0.01^e^	8.90 ± 0.02^a^	2.91 ± 0.01^c^	4.54 ± 0.02^c^
	DOD-K	3.56 ± 0.01^d^	2.98 ± 0.02^d^	1.79 ± 0.02^e^	2.10 ± 0.02^e^
**Accra**	AMZ-D	6.19 ± 0.01^a^	4.77 ± 0.01^b^	3.45 ± 0.01^d^	2.27 ± 0.02^d^
	AMZ-12 h	4.41 ± 0.01^b^	-	-	-
	AMZ-24 h	4.28 ± 0.01^c^	7.92 ± 0.01^a^	4.98 ± 0.01^c^	5.72 ± 0.01^b^
	AMZ-M	4.04 ± 0.01^d^	-	-	-
	AMZ-Su	3.68 ± 0.01^f^	7.83 ± 0.02^a^	5.78 ± 0.01^b^	5.65 ± 0.01^b^
	AMZ-Sd	3.65 ± 0.01^f^	7.86 ± 0.02^a^	5.94 ± 0.01^a^	5.86 ± 0.02^a^
	AMZ-K	3.79 ± 0.01^e^	3.59 ± 0.02^c^	2.73 ± 0.01^e^	2.68 ± 0.01^c^

NB: Figures are presented as means of two samples ± SD and different superscript letters to figures are significantly different at *P* ≤ 0.05;—= not enumerated

^*^D = dry millet grains, 12 h, 24 h = after 12 or 24 h of steeping, M = milled steeped millet grains and spices, Su = supernatant of slurry, Sd = sediment of slurry, K = *Hausa koko*

### Changes in the population of LAB and Yeast during the production of *Hausa koko*

LAB and yeasts were enumerated in all timepoints of the fermentations produced by the different processors, except the 12 h steeping timepoint in samples that were steeped for a total of 24 h. The populations of LAB and yeasts during the production of *Hausa koko* are shown in Table 1. The LAB counts in the grains were log 3.18–4.79 CFU/g. At the end of the slurry fermentation, the LAB population had increased (*p*-value < 0.001) by four log units to log 7.64–8.94 CFU/g. In the cooked *Hausa koko,* the LAB population decreased to log 2.77–3.95 CFU/g (*p*-value < 0.001). Similar changes were observed for the *Lactococci*, although differences were not statistically significant (*p* = 0.054). The population of *Lactococci* was usually about half of the counts recorded for the LAB, though in a few instances, they were much higher. The same trends as for LAB were observed for yeasts (*p*-value = 0.0001). The corresponding yeast populations were log 2.02–3.88 CFU/g in the millet grains, log 4.54–6.98 CFU/g at the end of slurry fermentation and log 2.10–2.98 CFU/g in the *Hausa koko* samples.

### Characterisation and identification of lactic acid bacteria

The isolates grown on the selective media MRS and M17 agar plates which were Gram-positive, catalase-negative, and oxidase-negative were assumed to be LAB. They were mostly rods and occurred in singles, pairs, or chains. Isolates were confirmed as single species by bacterial colony PCR of the 16S rRNA gene and typed using (GTG)5 sequence-based rep-PCR. The rep-PCR gel images were used to select LAB isolates that stood out as distinct from one another.

Out of 500 LAB isolates, a total of 70 were chosen, whole genome sequenced and submitted to NCBI. Nine different LAB species were identified: *Limosilactobacillus pontis* (31.4% of the sequenced isolates), *Pediococcus acidilactici* (20.0%), *Limosilactobacillus fermentum* (17.1%), *Limosilactobacillus reuteri* (14.3%), *Pediococcus pentosaceus* (4.3%), *Lacticaseibacillus paracasei* (4.3%), *Lactiplantibacillus plantarum* (4.3%), *Schleiferilactobacillus harbinensis* (2.9%) and *Weissella confusa* (1.4%).

**Fig. 1:**
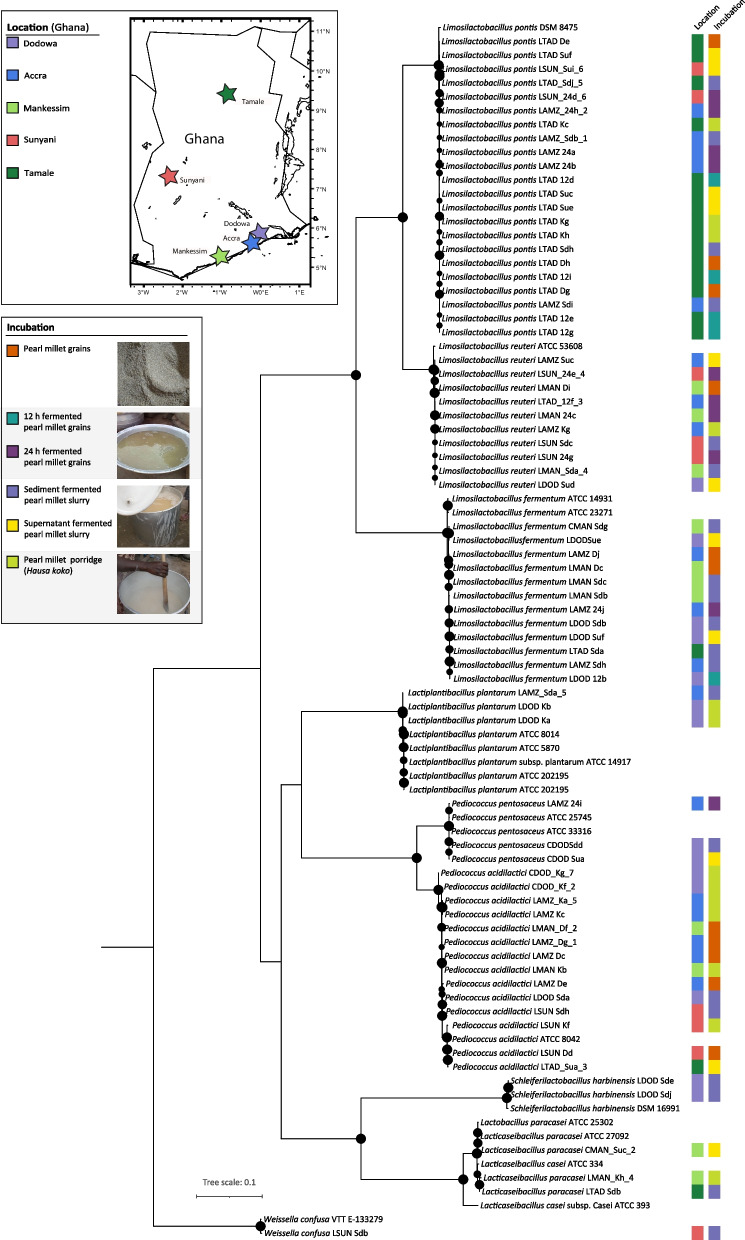
Phylogenomic tree reconstruction of bacterial isolates obtained from the fermentation process. Circles in the partitions represent partitions with > 75 bootstrap support and the size is proportional to the support. The map of Ghana was visualized with GeoMapApp V.3.6.15 and edited with Illustrator

**Fig. 2:**
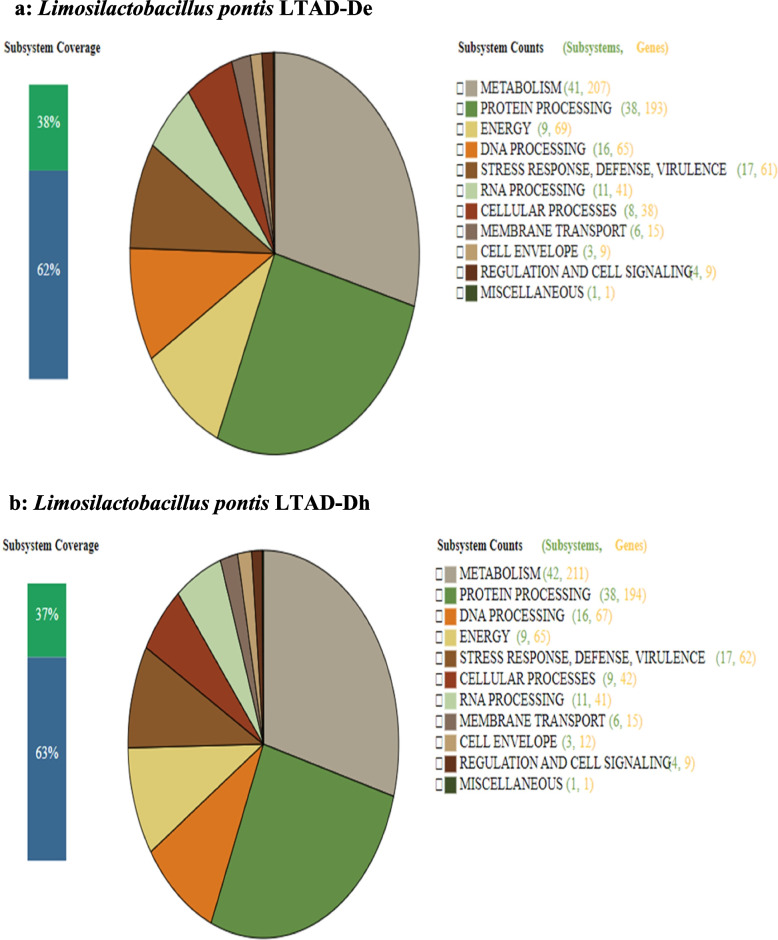
**a** and **b **Subsystem characterisation of two *L. pontis* strains from the same production site (Tamale). The green/blue bar shows the subsystem coverage in percentage

Phylogenetic assignment of the LAB genome assemblies showing the different species that were identified at the various production sites, and processing stages or time points are shown in Fig. 1. The subsystem analysis predicted by PATRIC (http://patricbrc.org) database v3.6.2. showed that despite the nucleotide similarities between all isolates of the same species, their metabolic features were dissimilar and had different metabolic capabilities. This indicated that different strains of the same species could be present in the same sample. For example, isolates *Limosilactobacillus pontis* LTAD-De and *Limosilactobacillus pontis* LTAD-Dh from the same production site and time point show a different subsystem profile (Fig. 2a and b). The green bar of the subsystem coverage corresponds to the percentage of the proteins included in the subsystems while the blue bar corresponds to the percentage of the proteins that are not included in the subsystems [50, 51].

### The proportions of LAB species occurring during the production of *Hausa koko*

Table 2 shows the frequency at which different species of LAB were isolated during the production (i.e., at various stages of processing of millet into *Hausa koko*) of *Hausa koko* in several towns/districts. For each of the locations, the figure given is the percentage of the LAB species in all LAB isolates taken at the various stages of production from all the production sites in the town/district. At Dodowa, LAB isolates identified by whole genome sequencing from the various stages of production of *Hausa koko* at the different production sites in order of predominance were *L. fermentum* (28.56%)*, P. acidilacti* (21.43%), *P. pentosaceus* (14.29%), *S. harbinensis* (14.29%), *L. plantarum* (14.29%), and *L. reuteri* (7.14%)*. L. paracasei* and *L. pontis* which were isolated in *Hausa koko* production in some of the other production sites/metropolises were not isolated at Dodowa. In the Tamale metropolis, the most frequently isolated LAB species in *Hausa koko* production was *L. pontis* which accounted for 78.96% of all the LAB isolates. At Sunyani, only four LAB species, *L. reuteri, P. acidilacti, L. pontis* and *W. confusa* were isolated, with *L. reuteri* and *P. acidilacti* accounting for more than 66% of all the LAB isolated from *Hausa koko* production. All the other five LAB species found in *Hausa koko* production were absent. At Mankessim the dominant LAB species isolated in *Hausa koko* production were *L. fermentum* (36.37%) and *L. reuteri* (27.27%). The other two LAB species isolated in addition to these were *P. acidilacti* and *P. paracasei.* In the Accra metropolis, the most frequently isolated LAB species in *Hausa koko* production were *L. pontis* (29.41%), *P. acidilacti* (29.41%) and *L. fermentum* (17.66%).

**Table 2:** Percentage (%) of LAB species identified at the different production sites involving all processing stages

LAB species	Percentage (%) of LAB species identified in different *Hausa koko* production sites				
	Dodowa	Tamale	Sunyani	Mankessim	Accra
*Limosilactobacillus fermentum*	28.56	5.26	-	36.37	17.66
*Limosilactobacillus reuteri*	7.14	5.26	33.33	27.27	11.76
*Weissella confusa*	-	-	11.11	-	-
*Pediococcus acidilacti*	21.43	5.26	33.33	18.18	29.41
*Limosilactobacillus pontis*	-	78.96	22.23	-	29.41
*Pediococcus pentosaceus*	14.29	-	-	-	5.88
*Lacticaseibacillus paracasei*	-	5.26	-	18.18	-
*Lactiplantibacillus plantarum*	14.29	-	-	-	5.88
*Schleiferilactobacillus harbinensis*	14.29	-	-	-	-

- = not isolated

Table 2 further shows that it was only *L. reuteri* and *P. acidilacti* that were isolated in all five districts/towns, whilst *L. fermentum* was isolated in four out of the five districts/towns. *L. pontis* was isolated in three out of the five districts/towns and *L. paracasei* in only two out of the five districts/towns. *P. pentosaceus, L. paracasei* and *L. plantarum* were only isolated in two out of the five districts/towns. *W. confusa* and *S. harbinensis* were isolated only at Sunyani and Dodowa respectively out of the five districts/towns. The presence and abundance of different taxa across regions could be attributed to the source of the grain, as well as different environmental and processing conditions. The microbiota of the different geographical sites will be of importance for the selection and designing of a starter culture in future studies.

### The composition of lactic acid bacteria at different stages of *Hausa koko* production

The composition of the LAB population at different stages of *Hausa koko* is presented in Table 3. All the microorganisms that occurred in the millet grains were present at all the processing stages at varying percentage occurrences except for *P. acidilactici* in the steeped millet samples (12 and 24 h). *P. acidilactici* and *L. pontis* were prominent and remained the dominant species from the beginning till the end of the processing stages except for the dominance of *L. fermentum* in the sediment.

**Table 3:** Composition of LAB population at different stages of *Hausa koko* production at the five production sites

Processing Stages	LAB Species	Percentage (%) Occurrence
**Dry Grains**	*Pediococcus acidilactici*	45.46
11 Isolates, 4 strains	*Limosilactobacillus pontis*	27.27
	*Limosilactobacillus fermentum*	18.18
	*Limosilactobacillus reuteri*	9.09
**12 & 24 h**	*Limosilactobacillus pontis*	57.15
14 Isolates, 4 strains	*Limosilactobacillus reuteri*	28.57
	*Limosilactobacillus fermentum*	7.14
	*Pediococcus pentosaceus*	7.14
**Supernatant**	*Limosilactobacillus pontis*	36.37
11 Isolates, 6 strains	*Limosilactobacillus fermentum*	18.18
	*Limosilactobacillus reuteri*	18.18
	*Pediococcus pentosaceus*	9.09
	*Lacticaseibacillus paracasei*	9.09
	*Pediococcus acidilactici*	9.09
**Sediment**	*Limosilactobacillus fermentum*	30.00
19 Isolates, 9 strains	*Limosilactobacillus pontis*	20.00
	*Limosilactobacillus reuteri*	10.00
	*Pediococcus acidilactici*	10.00
	*Schleiferilactobacillus harbinensis*	10.00
	*Lacticaseibacillus paracasei*	5.00
	*Weissella confusa*	5.00
	*Pediococcus pentosaceus*	5.00
	*Lactiplantibacillus plantarum*	5.00
***Hausa koko***	*Pediococcus acidilactici*	42.87
14 Isolates, 6 strains	*Limosilactobacillus pontis*	21.43
	*Lactiplantibacillus plantarum*	14.28
	*Limosilactobacillus fermentum*	7.14
	*Limosilactobacillus reuteri*	7.14
	*Lacticaseibacillus paracasei*	7.14

In the supernatants, *L. pontis* dominated whilst in the sediments *L. fermentum* was dominant. Given that they are both a part of the same time point, the supernatant and sediment had the same array of LAB except for the occurrence of *S. harbinensis*, *W. confusa* and *L. plantarum* in the sediment which were absent in the supernatant. In the final *Hausa koko* samples, *P. acidilactici* and *L. pontis* were the dominant LAB. *L. pontis, L. fermentum* and *L. reuteri* were the only LAB species that were isolated at all the different stages of the *Hausa koko* production process with varied percentage occurrence, although only *L. reuteri* was isolated at all the production sites. *P. acidilactici* and *P. pentosaceus* occurred in four and three processing stages respectively at varying percentages.

### Yeasts involved in *Hausa koko* fermentation

**Fig. 3:**
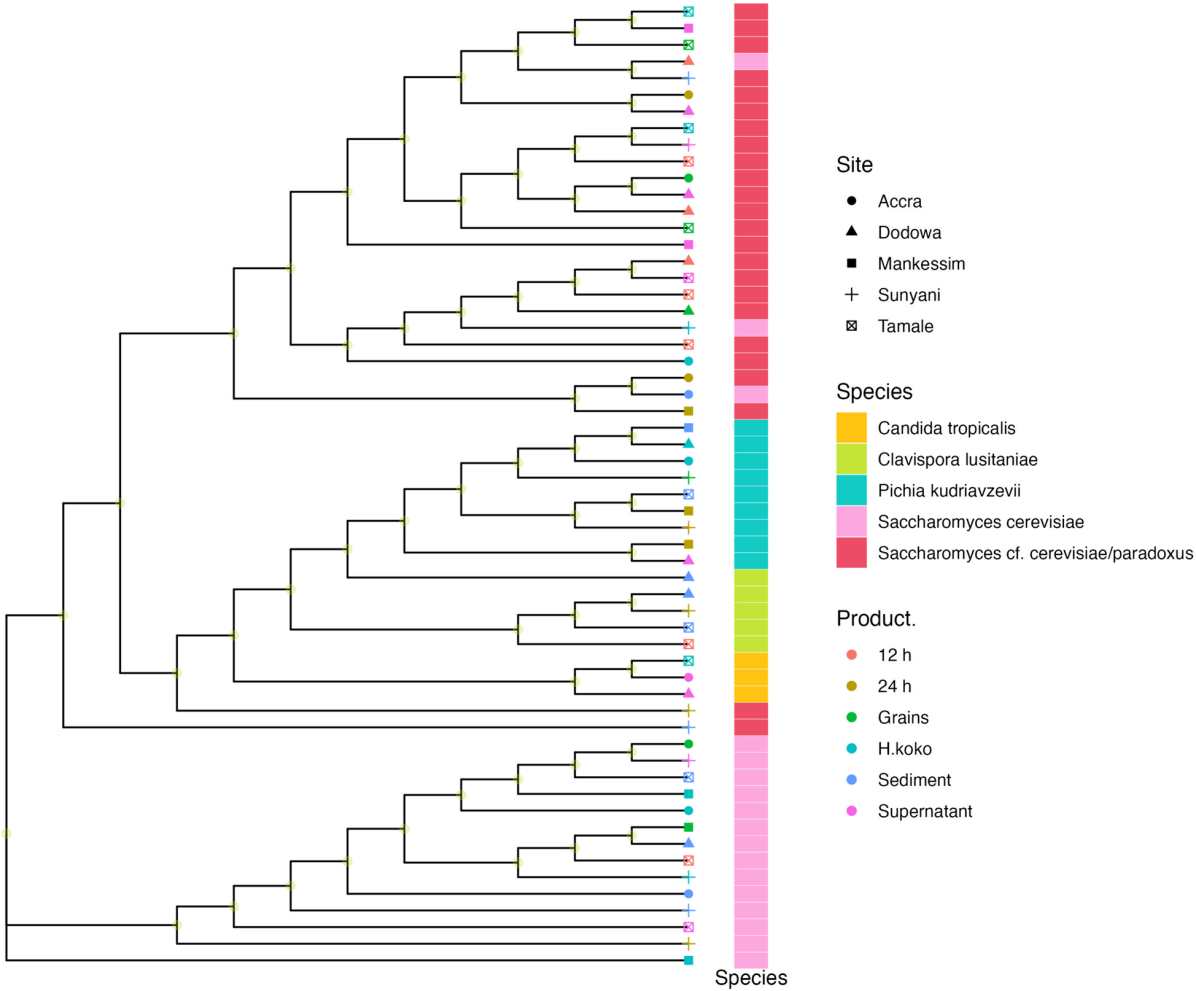
Phylogenetic assignment of the yeast species identified from the various production sites the samples were collected from i.e., Tamale, Accra, Sunyani, Mankessim, and Dodowa

For the yeast isolates, 58 out of 250 isolates were randomly selected and identified using the NCBI database as *Saccharomyces cf. cerevisiae/paradoxus* (41.4%), *Saccharomyces cerevisiae* (31.0%), *Pichia kudriavzevii* (13.8%)*, Clavispora lusitaniae* (8.6%) and *Candida tropicalis* (5.2%). These percentages represent the total yeast species isolated, in all sites and at all stages. The 28S rRNA gene sequences showed 99 -100% identity to identified species. Phylogenetic assignment of the 28S rRNA gene Sanger sequencing of yeast species identified at the different production sites and processing stages are shown in Fig. 3. The type and percentage occurrence of the yeast from the different production sites is shown in Table 4.

**Table 4:** Identification and occurrence of yeast at the different production sites

Production Site	Yeast Species	Percentage (%) Occurrence
**Dodowa**	*Saccharomyces cf. cerevisiae/paradoxus*	53.85
	*Saccharomyces cerevisiae*	23.08
	*Candida tropicalis*	15.38
	*Clavispora lusitaniae*	7.69
**Tamale**	*Pichia kudriavzevii*	38.46
	*Saccharomyces cerevisiae*	23.08
	*Saccharomyces cf. cerevisiae/paradoxus*	23.08
	*Clavispora lusitaniae*	7.69
	*Candida tropicalis*	7.69
**Sunyani**	*Saccharomyces cerevisiae*	50.00
	*Saccharomyces cf. cerevisiae/paradoxus*	41.67
	*Clavispora lusitaniae*	8.33
**Mankessim**	*Saccharomyces cerevisiae*	44.45
	*Pichia kudriavzevii*	22.22
	*Saccharomyces cf. cerevisiae/paradoxus*	22.22
	*Clavispora lusitaniae*	11.11
**Accra**	*Saccharomyces cf. cerevisiae/paradoxus*	60.00
	*Pichia kudriavzevii*	20.00
	*Saccharomyces cerevisiae*	10.00
	*Clavispora lusitaniae*	10.00

The most frequently isolated yeast species from the *Hausa koko* production sites was *S. cf. cerevisiae/paradoxus.* In addition to *S. cf. cerevisiae/paradoxus,* the 28S rRNA gene sequencing also identified some isolates as *S. cerevisiae.* Both were associated with the fermentation of millet in *Hausa koko* production at all the production sites.

*P. kudriavzevii* was the third most dominant yeast (13.8%) of the total yeast isolated in *Hausa koko* production. It was isolated at the Tamale, Mankessim, and Accra production sites.

*C. lusitaniae* (8.6%) and *C. tropicalis* (5.2%) were the other yeast species identified and were present in low numbers. Although they were not the predominant species, *C. lusitaniae* was isolated at all the production sites whilst *C. tropicalis* was isolated only at Tamale and Dodowa sites.

*S. cf. cerevisiae/paradoxus, S. cerevisiae, C. tropicalis,* and *C. lusitaniae* were the yeast species that occurred in the millet grain samples. The subsequent production stages all recorded four yeast species each at varying percentage occurrences. At the steeping (12 and 24 h) and sediment stages, *C. tropicalis* was replaced by *P. kudriavzevii* whilst *C. lusitaniae* was also replaced by *P. kudriavzevii* at the supernatant and *Hausa koko* stages. *S. cf. cerevisiae/paradoxus* dominated the grains (42.86%) and steeping (12 and 24 h) stages (64.71%) whilst *S. cerevisiae* dominated the supernatant (45.45%) and sediment stages (41.67%). Their dominance was however overtaken by *P. kudriavzevii* in the final product (45.45%).

## Discussion

### Lactic acid fermentation of *Hausa koko*

The reductions in pH during *Hausa koko* production at the various stages and production sites were significantly different. This may be attributed to the variations and composition of the different substrates, different LAB profiles and populations. An increase in the population of LAB may produce acidic metabolites that lower the pH [2, 11]. As the pH reduced, the population of LAB and yeast increased in the fermentation stages (12 to 24, Su and Sd) but reduced in the final porridge which may be attributed to the application of heat [2]. Production of sour food products involving an increase in lactic acid population and a decrease in pH is characteristic of fermented products. In Ghana, this trend has been reported in different fermented foods [19, 20, 52, 53]. In Nigeria, Sherifah and Daodu (2011) reported a reduction in pH from 5.7 to 3.5 during *ogi* production from maize [54]. In Benin, Houngbédji et al. (2018) reported reductions from mean values of 5.4 at 0 h to 4.1 at 36 h of fermentation during *mawè* production [14]. The low pH resulting from the lactic acid production of *Hausa koko* contributes to its organoleptic quality as well as safety as a food product.

Spontaneously fermented cereal foods often exhibit microbial successions [14]. Different species of lactic acid bacteria and yeasts were isolated at the different stages of *Hausa koko* production and at the different production sites. The diversity of lactic acid bacteria encountered at the different stages of *Hausa koko* production is likely to have originated from the raw materials and processing equipment as suggested by [55] regarding yeast sources in the fermentation of African indigenous foods with reference. There was a steady increase in the population of LAB by 4 log units during the soaking of the millet grains through to the end of the fermentation of the millet slurry which had separated into a supernatant and sediment. The LAB phylogenomic tree showed a consistent grouping per species, as expected. However, differences were observed even within the same species, indicating the existence of different strains with different metabolic capabilities [56].

In the present work, the most frequently occurring LAB responsible for the fermentation of millet grains and millet slurry during *Hausa koko* production were *L. pontis*, *L. fermentum, L. reuteri, P. pentosaceus, P. acidilactici,* and *L. paracasei*. These results are similar to the findings of [57] who identified *L. fermentum, W. confusa, Pediococcus spp,* (*P. acidilactici* and *P. pentosaceus*) and *L. salivarius* as LAB responsible for *Hausa koko* fermentation in the Tamale municipality based on the sequencing of the 16S rRNA gene. In the present work, *Lactobacillus salivarius* was not isolated in *Hausa koko* fermentation, however, a larger number of LAB species were encountered, including *L. pontis*, *L. reuteri, L. paracasei,* and *S. harbinensis*. In this study, more LAB species were identified at each processing stage than was reported by [55]. It is important to note that *Limosilactobacillus fermentum* and *Lactobacillus fermentum* are the same organisms following the reclassification of the genus *Lactobacillus* [58].

Two reasons may account for the additional species reported in the present work. Firstly, samples were taken from five different locations in five regions which represents a wider geographical area in comparison to the work of [57] whose samples were taken from only one of the regions, Northern (Tamale). Also, in the present study, the LAB isolates were identified by whole genome sequencing which has a higher discriminatory power in distinguishing between different species as compared to sequencing with the 16S rRNA gene reported in the previous study [57]. *L. pontis* was identified in three out of five production sites located in Tamale, Sunyani, and Accra, though it had not previously been reported in traditional food fermentation in Ghana. *L. pontis*, which was identified either as the most dominant (12 and 24 h, supernatant) or next dominant (dry millet grains, sediment and *Hausa koko* stages) LAB in the overall processing of *Hausa koko* production in the present study, has also been reported to be associated with sourdough fermentation [59, 60]. It is also associated with the spontaneous fermentation of Ethiopian non-alcoholic cereal beverages, *borde* [61], and *mursik* fermented milk from Kenya [62].

Two LAB were isolated in all five production sites: *L. reuteri* and *P. acidilactici*. Both bacteria are heterofermentative, meaning they produce not only lactic acid but also ethanol, acetic acid and CO_2_ as by-product of glucose fermentation, in contrast to homofermentative LAB which produces only lactic acid as by-product. *L. reuteri* normally resides in the gastrointestinal tract of humans and animals and has the capability to produce organic acids, ethanol, and enzymes. It can secrete the antimicrobial reuterin which is stable at a large range of pH values, bile salt hydrolase, lipolytic and proteolytic enzymes. It can target and control the growth of both Gram-positive and Gram-negative spoilage and pathogenic bacteria in foods. It can stably colonize the mammalian intestine and benefit the immune system of the host. *L. reuteri* also produces vitamins and other antimicrobial substances that allow it to compete against pathogenic microbes [63–68]. *P. acidilactici* has antagonistic activities against some Gram-positive and Gram-negative organisms. It works in conjunction with lactic and acetic acid produced with possible protection against diseases in the gastrointestinal tract [69]. *P. acidilactici* is common in fermented dairy, meat, and vegetable products and some strains produce the antimicrobial pediocin which also inhibits several spoilage and pathogenic organisms. They have been used as flavour enhancers due to the formation of volatile compounds during milk fermentation in cheese production [70–73]. *P. acidilactici* has been reported in several indigenous African fermented foods [16, 74–76] and used in isolation or combination with other LAB in starter culture development [77–79].

The heterofermentative *L. fermentum* was isolated in four out of the five production sites and is one of the dominant LAB in *Hausa koko* production. We have previously reported *L. fermentum* to be one of the taxonomic groups explaining differences in microbial diversity between Hausa koko fermentation time points and production regions [2]. Lei et al. (2014) also reported *L. fermentum* to be predominant in millet fermentation to produce *Hausa koko*. *L. fermentum* has been reported widely in the fermentation of other cereals in Africa [57]. These include *doklu* [16], *ogi* [80], *kunun-zaki* [81], *nsiho* [19], *burukutu* [20], *mahewu* [82], *dolo* and *pito* [83] and several others.

*P. pentosaceus* was isolated at two of the production sites and three processing stages or time points. *P*. *pentosaceus* is homofermentative has antimicrobial and antioxidant properties, and is often used as a starter culture bacterium for fermenting foods with good bio-preservation characteristics [84–86]. *P*. *pentosaceus* can tolerate low pH/acids and bile salts, improve safety and quality, extend shelf life, has anti-mycotoxin effect, and affect the flavour characteristics of food products [84–86]. This bacterium has been associated with the fermentation of cereal-based foods such as *borde* from Ethiopia [61] and *dèguè* from Burkina Faso [87]. It was also isolated from *omegisool*, a traditional Korean fermented millet alcoholic beverage and exhibited resistance to different antibiotics, adhesion capacity, and antioxidant activity [88].

*W. confusa*, which is heterofermentative, was isolated in only one out of the five production sites and is associated with a variety of fermented foods such as *mawè* [14, 89, 90]. Several strains of *W. confusa* have been established as probiotics in nature, mainly because of their antimicrobial properties, with few strains identified as opportunistic bacteria. They have been proposed as a probiotic starter culture due to their inhibitory ability and antifungal activity [91, 92]. Houngbédji et al*.*, (2018) reported the occurrence of *W. confusa* mainly at the onset of a cereal-based food *mawè,* fermentation in Benin [14]. In this study, although *W. confusa* was isolated in low numbers, its occurrence at a production site indicates its association with *Hausa koko* fermentation as reported by [57]. It has been associated with other fermented pearl millet foods including *fura* and *Kimere* [31, 93].

*L. paracasei* was isolated at the Tamale and Mankessim production sites whilst *S. harbinensis* (formally *L. harbinensis*) and *L. plantarum* were isolated only at the Dodowa site. *L. plantarum* and *L. paracasei* subsp. *paracasei* have been reported in *bushera* in Uganda [94]. *L. pentosus*, *L. plantarum,* and *L. paraplantarum* share similar phenotypic characteristics and similar 16S rRNA gene sequences (≥ 99%) which makes it difficult to differentiate between them except by WGS [95]. *L. plantarum* has been reported in the fermentation of maize, millet, and sorghum in the production of *akamu* and *kunu-zaki* [96]. The presence of *L. paraplantarum* was reported at the initial stages of millet fermentation during *fura* production in Ghana by Owusu-Kwarteng et al*.*, (2012). Facultative heterofermentative *S. harbinensis* has been reported in sorghum sourdough fermentation [97], and *S. harbinensis, L. plantarum*, and *L. paracasei* in raw milk and cheese fermentation [98].

### Involvement of yeast in *Hausa koko* fermentation

LAB and yeast occur naturally in the ecological niche of cereals and play significant roles during their fermentation [59]. The presence of yeasts has been reported in several fermented foods and their relationship with LAB in such fermentations has been established [11, 16] reported similar LAB and yeast counts during the fermentation of maize flour during *doklu* production, where LAB and yeast increased from log 4.2 to 9 CFU/g and log 4.9 to 7.8 CFU/g respectively. The increasing trend in the yeast population can be attributed to their great growth rate compared to other microorganisms [99]. In the present study, the yeast population during *Hausa koko* production was dominated by *S. cf. cerevisiae/ paradoxus* and *S. cerevisiae*. They accounted for about 70% of the total yeast population in *Hausa koko* production and were found at all five production sites located in the five different geographical regions of Ghana. This is in accordance with our previous report that the fungal community during *Hausa koko* fermentation was dominated by the genus *Saccharomyces* [2]*. S. paradoxus* is the closest known species to *Saccharomyces cerevisiae* [100, 101]*.* The genome of *S. paradoxus* is highly conserved when compared to *Saccharomyces cerevisiae.* In coding regions, the genome of *S. paradoxus* shares 90% of its identity with the genome of *S. cerevisiae*, and in the intergenic regions, it has 80% homology [102]. *S. paradoxus* is the undomesticated relative of *Saccharomyces cerevisiae* [100, 103]. They co-exist in a similar environment. *Saccharomyces paradoxus* is almost morphologically indistinguishable from *Saccharomyces cerevisiae* in nearly all aspects of morphology, metabolism, and its life cycle [104]. This could be seen by the phylogenetic analysis of yeast isolates using 28S rRNA gene Sanger sequencing, which revealed that these isolates clustered in specific groups, demonstrating their phylogenetic relatedness.

The yeast population in most African fermented cereal foods has also been reported to be dominated by *S. cerevisiae.* These include *mawè* [14], *ogi* [105], cereal-based fermented foods [4], *burukutu* [20], and many others [4, 106]. In contrast, *S. paradoxus* has only been reported in a few instances: in *akamu*, a cereal-based complementary food [107], and sorghum beer from Ghana and Burkina [108]. It is noted that in the two instances where the presence of *S. paradoxus* was reported in the African traditional foods, the authors used molecular characterisation involving sequencing of the internal transcribed spacer regions (ITS1 and ITS2). It is, therefore, possible that in some of the instances where *S. cerevisiae* has been reported and identification was by phenotypic characterisation based mainly on the fermentation and utilization of different sugars, the yeasts could have been *S. paradoxus*. This is because they co-exist, share the same phenotypic characteristics and would be identified as *S. cerevisiae* using the API kit [104]. It is therefore likely that *S. paradoxus* plays a greater role in the fermentation of indigenous African fermented foods than has been reported.

The other yeasts found in *Hausa koko* production in the present work were *P. kudriavzevii, C. lusitaniae*, and *C. tropicalis. P. kudriavzevii* is the teleomorph of *Candida krusei* with a few strains being opportunistic pathogens [109]. The presence of *C. krusei/P. kudriavzevii* has been reported extensively in African fermented cereal and other foods including *mawe* [106], *gowe* [76], and *agbelima* [26]. *C. lusitaniae* and *C. tropicalis* have been reported in other fermented cereals in Africa. *C. lusitaniae* in *obushera* [110], *ogi* [23, 106], and *C. tropicalis* in *togwa* [75]. *Pichia, Candida*, *Kluyveromyces, Nakaseomyces, Torulaspora,* and *Cyberlindnera* were also among the other genera reported in our previous study on *Hausa koko* [2]. Yeasts cause acidification and produce ethanol, carbon dioxide, extracellular enzyme production, as well as generating flavour compounds and bio-preservatives [23, 111, 112].

Most of the LAB and yeast species characterised in the present study in *Hausa koko* are associated with many other indigenous African fermented foods and play important roles during the process with some deemed as potential probiotic species for starter culture development. It is therefore possible that these LAB and yeast species characterised in this study may also possess such characteristics and hence represent a valuable resource for future study.

## Conclusion

The central operation in the processing of millet into *Hausa koko* is fermentation, which involves the steeping of millet grains and spontaneous fermentation of the steeped grains that have been milled together with spices and made into a slurry. Fermentation in *Hausa koko* production has been confirmed to be an acidification process that involves the growth of LAB and yeasts, resulting in the lowering of pH. The pH reduced from a range of 6.02 to 6.53 in the grains to 3.51 to 3.99 in the final *Hausa koko* product*.* The predominant species of LAB responsible for the souring fermentation identified by whole genome sequencing were *Limosilactobacillus pontis, Pediococcus acidilactici*, *Limosilactobacillus fermentum* and *Limosilactobacillus reuteri.* The yeast species were identified to be *Saccharomyces cf. cerevisiae/paradoxus*, *Saccharomyces cerevisiae, Pichia kudriavzevii*, *Clavispora lusitaniae*, and *Candida tropicalis*. The lactic acid bacteria *Limosilactobacillus pontis* and *Schleiferilactobacillus harbinensis* and the yeast *Saccharomyces paradoxus* were found to be involved in the fermentation of millet during *Hausa koko* production in Ghana for the first time.

Food safety and security are major issues, particularly in low medium income countries. Fermented cereals are popular, cheap, sustainable and locally produced and form an important part of the diet in Africa, especially during weaning. The common presence of food-borne pathogens and mycotoxins, and the low content of essential nutrients, are key areas for improvement. Understanding the fermentation process will highlight stages for improvement or intervention, while the identification of key microbes can lead to the development of effective starter cultures to improve the safety and nutritional value of these foods. Fermented foods also provide both a potential source of novel microorganisms with unexplored gene functions and an opportunity to study microbial interactions within a complex changing microbiome. This study provided information about the predominant LAB and yeast populations in *Hausa koko* production and how they change during the fermentation process. The fully sequenced bacterial isolates and characterised yeasts can be used in future studies for controlled fermentation and the development of safer starter cultures, while functional analysis of the bacterial genomes may identify key functions of fermenting microbes. In addition, an understanding of the dynamic changes during the fermentation process can identify the best stages for starter culture addition or nutritional interventions.

## Data Availability

Data are available in the NCBI database: Accession Numbers for the yeast sequences are OR186448-OR186505 while bacterial genomes can be found under BioProject with accession number PRJNA932444.
